# Impact of Resident Rotations on Critically Ill Patient Outcomes: Results of a French Multicenter Observational Study

**DOI:** 10.1371/journal.pone.0162552

**Published:** 2016-09-14

**Authors:** Benjamin G. Chousterman, Romain Pirracchio, Bertrand Guidet, Philippe Aegerter, Hervé Mentec, CUB-REA network

**Affiliations:** 1 Service de Réanimation Polyvalente, Centre Hospitalier Victor Dupouy, Argenteuil, France; 2 Service d'Anesthésie-Réanimation, Hôpital Européen Georges Pompidou, Université Paris Descartes, PRES Sorbonne Paris Cité, AP-HP, Paris, France; 3 Centre de Recherche en Epidémiologie, Equipe ECSTRA, INSERM 1153, PRES Sorbonne Paris Cité, Paris, France; 4 Department of Anesthesia and Perioperative Care, San Francisco General Hospital, University of California San Francisco, San Francisco, California, United States of America; 5 Service de Réanimation Médicale, Hôpital Saint-Antoine, AP-HP, Paris, France; 6 Département d'Information Hospitalière et Santé Publique—Unité de Recherche Clinique, Hôpital Ambroise Paré, AP-HP, Boulogne, France; University of Pittsburgh, UNITED STATES

## Abstract

**Purpose:**

The impact of resident rotation on patient outcomes in the intensive care unit (ICU) has been poorly studied. The aim of this study was to address this question using a large ICU database.

**Methods:**

We retrospectively analyzed the French CUB-REA database. French residents rotate every six months. Two periods were compared: the first (POST) and fifth (PRE) months of the rotation. The primary endpoint was ICU mortality. The secondary endpoints were the length of ICU stay (LOS), the number of organ supports, and the duration of mechanical ventilation (DMV). The impact of resident rotation was explored using multivariate regression, classification tree and random forest models.

**Results:**

262,772 patients were included between 1996 and 2010 in the database. The patient characteristics were similar between the PRE (n = 44,431) and POST (n = 49,979) periods. Multivariate analysis did not reveal any impact of resident rotation on ICU mortality (OR = 1.01, 95% CI = 0.94; 1.07, p = 0.91). Based on the classification trees, the SAPS II and the number of organ failures were the strongest predictors of ICU mortality. In the less severe patients (SAPS II<24), the POST period was associated with increased mortality (OR = 1.65, 95%CI = 1.17–2.33, p = 0.004). After adjustment, no significant association was observed between the rotation period and the LOS, the number of organ supports, or the DMV.

**Conclusion:**

Resident rotation exerts no impact on overall ICU mortality at French teaching hospitals but might affect the prognosis of less severe ICU patients. Surveillance should be reinforced when treating those patients.

## Introduction

It is commonly believed that the quality of health care may decrease when residents are rotating [1]. However, the actual impact of resident rotation on patient outcomes remains under debate. This effect, previously described in the United Kingdom[2] and the USA[3], is often referred to as the the “July effect”. A recent systematic review confirms that year-end resident rotations may be associated with increased mortality and decreased efficiency of care [4]. However, because of the high heterogeneity of these data, the magnitude of this effect has not been precisely estimated.

The importance of the July effect might differ between specialties. We hypothesized that the impact of resident turnovers on intensive care might be larger. Only two studies have specifically focused on patients in intensive care unit (ICU) [5, 6]; these studies consistently reported no association between resident rotation and patient outcome. Nevertheless, these two studies may have been underpowered. Moreover, the July effect might be even more pronounced when new residents are recruited twice a year, as in France [7, 8], rather than once at the end of the year, as in the UK or the USA. The aim of the present study was to estimate the effect of bi-annual resident rotation on ICU outcomes in a large French cohort of critically ill patients.

## Methods

To assess the impact of resident turnover on ICU outcomes, we used data included in the Collège des Utilisateurs de Base de Données en Réanimation (CUB-REA database) [9, 10] between 1996 and 2010.

### CUB-REA

The CUB-REA network is a group of up to 38 ICUs in teaching hospitals in Paris (France) and its suburbs. Its steering committee is composed of nine medical doctors and a database administrator (P.A.). At all participating ICUs, data from all consecutive patients are prospectively collected using a standardized web-based case report form. Each center is responsible for the completeness of the data. External quality controls are performed on a regular basis. The CUB-REA network and database were approved by the Commision Nationale de l’Informatique et des Libertés (French Watchdog Privacy Authority) (agreement #564407). Patient records/information were anonymized and de-identified prior to analysis

### Data Collected

In France, resident rotation occurs twice a year [7, 8] simultaneously throughout the country (at the beginning of November and at the beginning of May). Resident rotations encompass both resident rotations (residents advanced in their curriculum joining a department) and the arrival of new residents (first or second semesters in France). We recorded the exact rotation dates during the 1996–2010 period and then defined two periods: the period corresponding to the first month following the rotation was referred to as the POST period; the period corresponding to the fifth month of the rotation was referred to as the PRE period. The PRE period was not defined as the month immediately before the rotation to avoid any crossover between periods.

For each patient, demographic characteristics such as age, gender, comorbidities, admission diagnoses for ICU admission, admission modalities (direct or secondary admission), the modified Charlson comorbidity index [11, 12], and the Simplified Acute Physiology Score II (SAPS II) [13] score were collected. The intensity of ICU care was evaluated based on the number and type of organ supports provided during the ICU stay. The following therapies/monitoring devices were considered: mechanical ventilation (either invasive or non-invasive), catecholamine infusion, renal replacement therapy, and intracranial pressure monitoring. For all relevant variables, the percentage of missing data was less than 1%.

### Study Goals and Endpoints

The primary outcome was ICU mortality. The secondary outcomes were the length of stay in the ICU (LOS), the duration of mechanical ventilation (DMV), and the number of organ supports. The primary goal of this study was to assess the impact of the rotation period (POST versus PRE) on the specified endpoints.

### Statistical Analysis

Continuous variables are reported as means and standard deviations (SDs) or as medians and 25^th^-75^th^ percentiles as appropriate. The categorical variables are presented as counts and percentages (%). Comparisons were performed using Wilcoxon and Fisher’s exact tests as appropriate. The impact of resident rotation was initially estimated using mixed-effect multivariate logistic regression models adjusted for age, reason for ICU admission, number of days in the hospital before ICU admission, season, SAPS II [13], modified Charlson comorbidity index [11, 12], the number of organ failures, the occurrence of shock/ARDS, the interaction between the period and the season (fixed effects), and the center (random effect). Adjusted Cox proportional hazard models were used to fit the LOS or DMV values. The number of organ supports was analyzed using a generalized linear model.

To account for potential complex interactions between the variables, we used a non-parametric classification tree-based approach for variable importance measure. The association between ICU mortality and the potential predictors was explored using recursive partitioning (tree package for R [14]) with pruning to prevent over-fitting [15]. We used a random forest model [16] (RandomForest package for R [15, 17]) to quantitatively estimate variable importance based on a permutation accuracy importance measure^,^[15, 16]. To select the predictors of interest, we used a conservative decision rule, as suggested by Strobl et al. [15]. In addition, we quantitatively estimated the contribution of each potential predictor to the prediction of ICU mortality using the z-scores together with their p-values [15].

Sensitivity analyses were conducted by assessing the impact of resident rotations on different strata of severity as defined by the SAPS II. Thresholds were based on the 25^th^ and 75^th^ percentiles of the SAPS II distribution in our cohort.

All analyses were performed using R 2.15.1 statistical software (The R Foundation for Statistical Computing, Vienna, Austria) on a Mac OS X platform.

## Results

### Patient Characteristics

Thirty semesters, corresponding to 30 resident rotations, were analyzed. During this period (1996–2010), 262,772 patients were admitted to the 38 ICUs: 44,431 patients during the PRE period (the fifth month of the resident rotation) and 42,979 patients during the POST period (the first month of the resident rotation). The patient characteristics are summarized in Table 1, and the 38 participating ICUs are described in S1 Table. The median patient age was 59 [43–73. The median SAPS II at ICU admission was 36 [23–52]. The patient characteristics were similar between the PRE and POST periods (Table 1).

**Table 1:** Patient characteristics.

	Overall Population (n = 262,772)	Period POST (n = 42,979)	Period PRE (n = 44,431)	Rest of the semester (n = 219,793)
**Age**	59 [43.9–73]	59 [43.3–73.1]	59 [43.1–73]	59 [44–73]
**Septic shock**	26,854 (10.2%)	4,259 (9.9%)	4,434 (10%)	22,595 (10.3%)
**ARDS**	16,684 (6.3%)	2,641 (6.1%)	2,772 (6.2%)	14,043 (6.4%)
**Polytrauma**	946 (0.4%)	158 (0.4%)	146 (0.4%)	788 (0.4%)
**Charlson score**	0 [0–2]	0 [0–2]	0 [0–2]	0 [0–2]
**Number of organ failure**	1 [0–2]	1 [0–2]	1 [0–2]	1 [0–2]
**First SAPS II**	36 [23–52]	35 [22–52]	36 [23–52]	36 [23–52]
**Season: Winter**	135,631 (51.6%)	21,221 (49.4%)	23,226 (52.3%)	114,410 (52.1%)
**ICU LOS**	3 [2–7]	3 [2–7]	3 [2–7]	3 [2–7]
**Hospital LOS**	11 [4–24]	11 [4–24]	12 [4–25]	11 [4–24]
**Hospital LOS before ICU admission**	1 [1–2]	1 [1–2]	1 [1–2]	1 [1–2]
**Vasopressors**	79,608 (30.3%)	12,754 (29.7%)	13,320 (30%)	66,854 (30.4%)
**Mechanical Ventilation**	130,752 (49.8%)	21,018 (48.9%)	22,009 (49.5%)	109,734 (49.9%)
**Hemofiltration**	9,948 (3.8%)	1,543 (3.6%)	1,622 (3.7%)	8,405 (3.8%)
**Hemodialysis**	19,015 (7.2%)	3,030 (7%)	3,288 (7.4%)	15,985 (7.3%)
**ICP**	210 (0.1%)	36 (0.1%)	33 (0.1%)	174 (0.1%)
**ICU Death**	48,322 (18.4%)	7,872 (18.3%)	7,965 (17.9%)	40,450 (18.4%)

ARDS: acute respiratory distress syndrome; Charlson: modified Charlson comorbidity index; SAPS II: Simplified Acute Physiology Score II; ICU: intensive care unit; LOS: length of stay; ICP: intracranial pressure monitoring.

### ICU Mortality

**Fig 1:**
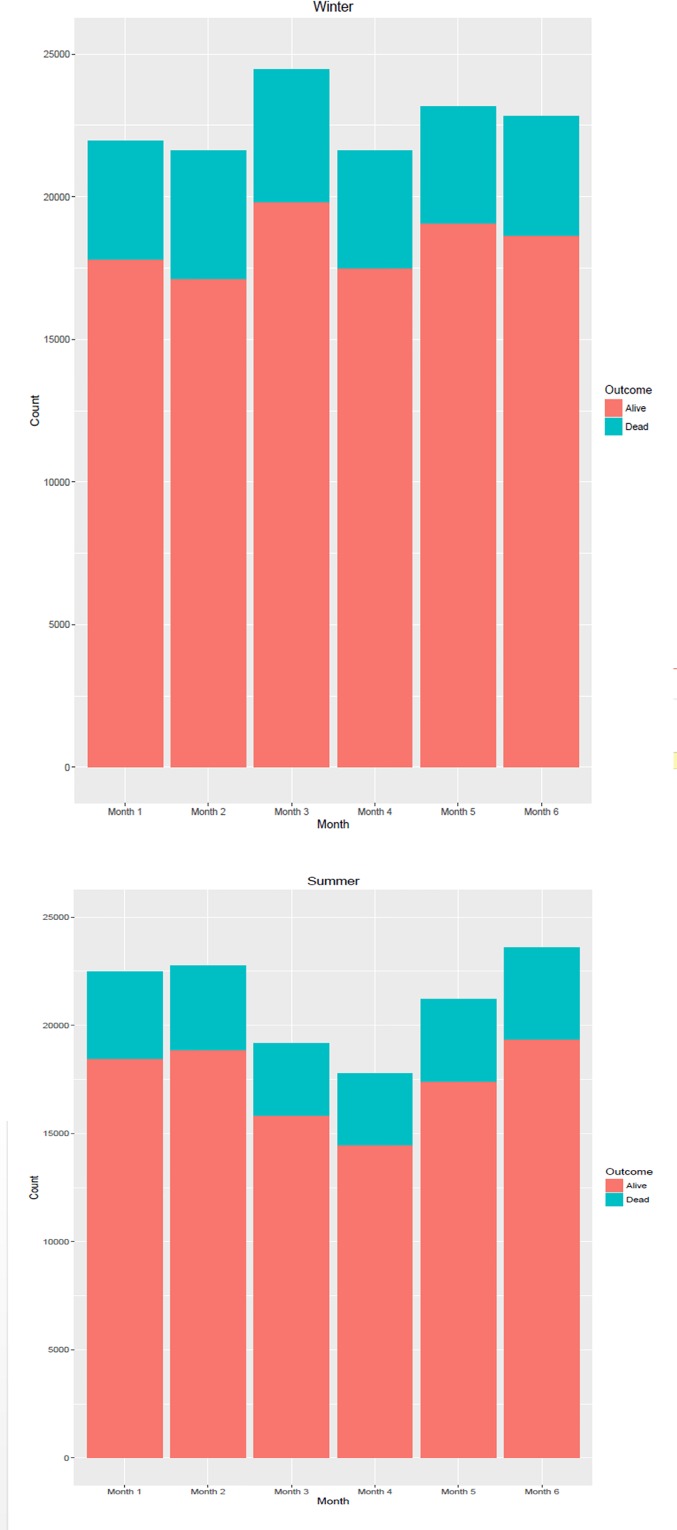
Monthly Survival Rate during a Resident semester. Upper panel: winter semester; lower panel: summer semester.

The average ICU mortality rate was 18.4% (95%CI = 18.2; 18.5). In the mild (SAPS II < 24), moderate (SAPS II between 24 and 52), and high severity (SAPS II > 52) groups, the average ICU mortality rate was 2.4% (95%CI = 2.3; 2.5), 9.3% (95%CI = 9.2; 9.5), and 53.5% (95%CI = 53.1; 53.9), respectively. Within rotations, the ICU admission and mortality rates were found to fluctuate over time (Fig 1).

**Fig 2:**
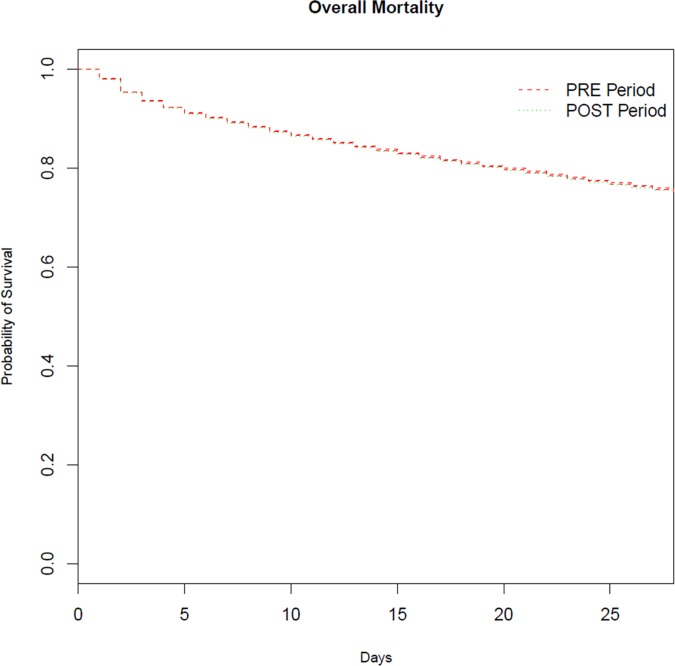
Survival Plots. Solid line: PRE period, dashed line: POST period.

No significant difference in ICU mortality was observed between the PRE and the POST periods (PRE, 17.9%; POST, 18.3%; p = 0.14; Table 1). Kaplan Meier curves for 28-day mortality are provided in Fig 2. After adjusting for age, reason for ICU admission, number of days in the hospital before ICU admission, season, SAPS II, Charlson comorbidity index, number of organ failures, occurrence of shock/ARDS, center, and the interaction between the season and the rotation period, the POST period was not found to be associated with increased ICU mortality (OR = 1.01, 95%CI = 0.94; 1.07, p = 0.91).

**Fig 3:**
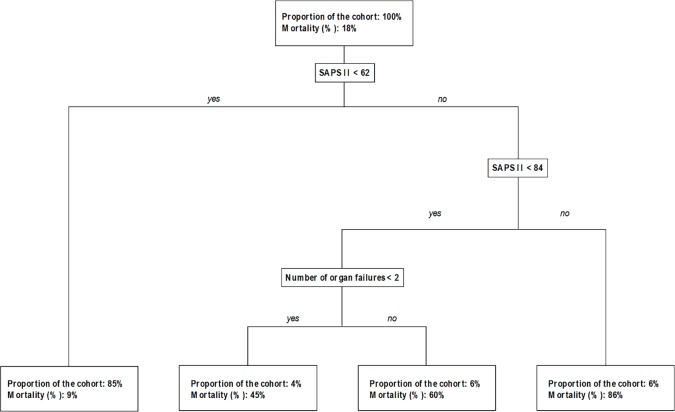
Single Classification Trees. Each node in the tree represents the splitting variable, as well as the splitting threshold for continuous variables.

**Fig 4:**
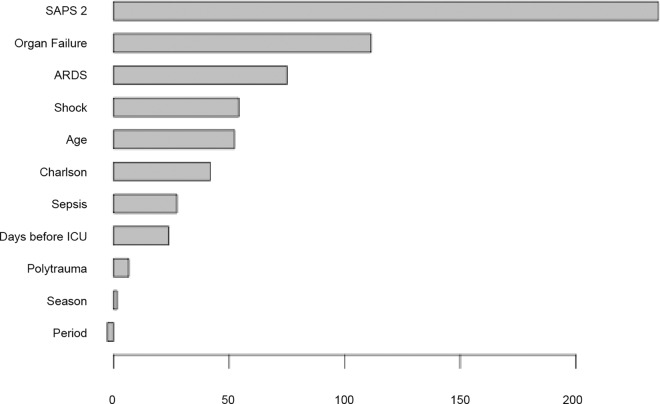
Permutation-Based Variance Importance Measure. The variables are ranked from the most important (top) to the least important (bottom). Variable importance is represented on the x-axis as the z-score. SAPS II: Simplified Acute Physiology Score II; ARDS: acute respiratory distress syndrome; Charlson: modified Charlson comorbidity index; ICU: intensive care unit.

We supplemented our analysis with recursive partitioning-based classification trees and a random forest model. The optimal tree obtained after pruning is presented in Fig 3. As illustrated in this figure, two predictors, the SAPS II and the number of organ failures, overtook all other potential explanatory variables. As demonstrated by a permutation-based variance importance measure from the random forest model (Fig 4), the value of the rotation period for predicting ICU mortality was found to be negligible. Moreover, the z-score corresponding to the rotation period, the SAPS II and the number of organ failures was -2.62 (p = 0.11), 235.70 (p = 0.009) and 111.44 (p < 0.001), respectively.

**Fig 5:**
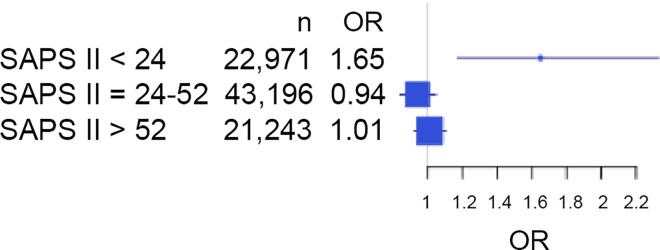
Impact of resident rotations on ICU Mortality According to Patient Severity. OR: odds ratio.

The impact of resident turnover varied across the severity strata (Fig 5). In the mild severity group (SAPS II < 24, n = 22,971), the POST period was associated with an increased ICU mortality (OR = 1.65, 95%CI = 1.17; 2.33, p = 0.004). However, the POST period was not associated with ICU mortality in the moderate (defined as a SAPS II between 24 and 52; n = 43,196, OR = 0.94, 95%CI = 0.84; 1.04, p = 0.25) or high severity (defined as a SAPS II score > 52) group (n = 21,243, OR = 1.01, 95%CI = 0.92; 1.10, p = 0.84).

### Secondary Outcomes

The median LOS was 3 [2–7] days for both the PRE and POST periods (p = 0.78). This result did not change after adjusting for potential confounders (HR = 1.01, 95%CI = 0.99; 1.02, p = 0.39).

The DMV was similar between the PRE and the POST periods (PRE period: mean = 3.4 days [min = 0; max 240] vs. POST period: mean = 3.5 [min = 0; max = 272]; HR = 1.01, 95%CI = 0.99; 1.02, p = 0.153). This result remained consistent after adjusting for potential confounders (HR = 0.99, 95%CI = 0.98; 1.01, p = 0.61).

The daily average number of organ supports per patient was slightly lower during the POST period than during the PRE period (PRE period: mean = 0.68 [min = 0; max = 11] vs. PRE period: mean = 0.71 [min = 0; max = 14]; risk difference (RD) = -0.15, 95%CI = [-0.28; -0.01], p = 0.040). The association between the number of organ supports and the rotation period was not significant after adjusting for potential confounders (RD = -0.08, 95%CI = [-0.21]; 0.04, p = 0.20).

## Discussion

In this large retrospective multicenter study in France, we found that biannual residents rotation exerts no significant impact on overall ICU mortality during the first month of the rotation. However, resident rotation was associated with an increase in ICU mortality in the less severe patients. The LOS, the DMV, and the number of organ supports were not significantly altered according to the rotation period.

The “July effect” has been extensively studied in various settings [3, 5, 6, 18–33], although the negative impact of new residents arrival remains under debate [2–4, 34–36]. Based on large cohort studies, some authors have reported associations between resident rotations, mortality rate, and adverse events [18, 23, 27, 32, 34, 37]. A recent systematic review [4] drew the conclusion that mortality increases and efficiency decreases in hospitals as a result of year-end changeovers. However, other results are conflicting [18–20, 28, 38–41], and several sources of heterogeneity might explain such discrepancies, especially the hospital type (teaching versus no teaching) or the intensity of care. The July effect is likely to be more pronounced in high-risk units such as ICUs. Accordingly, Jena et al. [27] recently reported increased mortality rates in patients hospitalized in July for acute myocardial infarction. However, Barry et al. [5] studied ICU patients admitted to a major teaching hospital and did not observe any association between resident rotation and patient outcomes. We hypothesized that assessing the impact of resident rotations would require a much larger database.

Based on standard multivariate regression analysis, we did not observe any significant adverse effect associated with new residents recruitment. However, this estimation may be biased because of the inability of the multivariate main term logistic regression model to capture complex interactions between predictors. Therefore, we also used a non-parametric modeling approach. Based on classification trees and a random forest model, we consistently found that the contribution of the resident turnover to ICU mortality was negligible. This result is in line with those concerning our secondary endpoints. We were unable to detect any associations between the residency period, the LOS, the DMV, or the number of organ supports, all of which are typically closely associated with ICU outcome. However, despite the absence of an effect on the overall population, resident rotation was associated with increased ICU mortality in the subgroup of patients with a SAPS II score < 24. There are several potential explanations for this result. First, when caring for the most severe patients, residents are likely to be more closely supervised by a senior physician but likely have greater autonomy when treating less severely ill patients. In addition, due to the high probability of death in severe ICU patients medical errors may be more likely to jeopardize the prognosis of a less severely ill patient. However, our results do not agree with those reported by Jena et al. [27] and Shuhaiber et al. [32], both of whom observed a more pronounced effect or resident rotation in high-risk patients. Nonetheless, the latter two studies focused on cardiac catheterization and cardiac surgery respectivelywhere the autonomy given to residents is typically more limited than that given to ICU residents.

Our study contains some limitations. First, it is a retrospective study. Therefore, some specific confounding factors may be missing and biasing the results. However, as illustrated in the tree analysis, ICU outcome is essentially driven by patient severity, which is well captured by the severity scores. French ICUs are described as closed units, i.e., units in which most medical decisions are made by attending intensivists rather than external consultants. Moreover, senior attending intensivists are present in the ICU every day and typically operate during night shifts. This particular organization of ICUs may limit the generalizability of our results to different health care systems. We chose to focus on the periods surrounding resident rotation dates. However, the rotation of other members of the medical staff may interfere with our results. For example, French fellows typically rotate after two years. Thus, when the new residents and the new fellows arrived simultaneously, the observed impact may have increased. However, we were unable to address this specific question in our study. Certain characteristics of the residents (previous experience in intensive care, etc.) are likely to interfere with the results. However, such information was not available. The data collection period was very long. We cannot avoid the confounders that ICU care and resident supervision have changed during this period. However, including time as a covariate in the models did not affect our results. Finally, the primary outcome was ICU mortality. Therefore, we cannot rule out a possible effect on subsequent outcomes such as readmission rate, 28-day mortality, and long-term health-related quality of life.

## Conclusion

In conclusion, we found that in France, bi-annual resident rotation exerts no impact on overall ICU mortality, the LOS, the DMV, or the intensity of ICU care. However, resident rotation may affect the prognoses of the less severe ICU patients. Thus, staff physicians should invest additional effort into supervising ICU care for such patients, especially at the beginning of each rotation.

### Key Messages

- Resident turnover does not impact overall ICU mortality
- Less severe patients are at risk of adverse outcome following resident turnover

## Supporting Information

S1 Table Description of the ICUs included in the study (DOCX)
