# Supplementary material for: Impact of Resident Rotations on Critically Ill Patient Outcomes: Results of a French Multicenter Observational Study
Source: PLoS One. 2016 Sep 14;11(9):e0162552. doi: 10.1371/journal.pone.0162552 (PMC5023104; doi:10.1371/journal.pone.0162552)
Supplement: S1 Table — (DOCX) [file pone.0162552.s001.docx]

**Electronic Supplemental Material**

**S1 Table.**

| **Center** | **N** | **Type** | **Number of beds** | **House senior staff** | **Number of residents** | **Resident/senior** | **University Hospital** |
| --- | --- | --- | --- | --- | --- | --- | --- |
| 1 | 6454 | MICU | 10 | 5 | 3 | 0.60 | Yes |
| 2 | 5768 | Both | 18 | 6 | 4 | 0.67 | No |
| 3 | 8961 | MICU | 12 | 5 | 3 | 0.60 | Yes |
| 4 | 10155 | Both | 16 | 7 | 3 | 0.43 | Yes |
| 5 | 1535 | MICU | 12 | 6 | 2 | 0.33 | Yes |
| 6 | 14483 | MICU | 20 | 6 | 5 | 0.83 | Yes |
| 7 | 4804 | MICU | 17 | 8 | 5 | 0.63 | Yes |
| 8 | 10653 | MICU | 18 | 6 | 4 | 0.67 | Yes |
| 9 | 16676 | MICU | 24 | 7 | 5 | 0.71 | Yes |
| 10 | 5072 | Both | 12 | 8 | 2 | 0.25 | No |
| 11 | 1034 | Both | 8 | 5 | 2 | 0.40 | No |
| 12 | 6647 | Both | 14 | 6 | 3 | 0.50 | No |
| 13 | 6835 | MICU | 20 | 6 | 4 | 0.67 | Yes |
| 14 | 489 | MICU | 12 | 7 | 3 | 0.43 | Yes |
| 15 | 6348 | Both | 12 | 4 | 2 | 0.50 | No |
| 16 | 13298 | MICU | 26 | 8 | 5 | 0.63 | Yes |
| 17 | 5222 | Both | 10 | 5 | 2 | 0.40 | No |
| 18 | 6334 | Both | 15 | 7 | 2 | 0.29 | No |
| 19 | 4911 | Both | 8 | 5 | 2 | 0.40 | Yes |
| 20 | 8527 | Both | 14 | 7 | 2 | 0.29 | No |
| 21 | 8474 | Both | 12 | 8 | 2 | 0.25 | Yes |
| 22 | 10863 | MICU | 15 | 5 | 4 | 0.80 | Yes |
| 23 | 4135 | Both | 10 | 6 | 2 | 0.33 | No |
| 24 | 6751 | Both | 10 | 6 | 2 | 0.33 | No |
| 25 | 1392 | SICU | 8 | 6 | 2 | 0.33 | No |
| 26 | 2443 | MICU | 12 | 6 | 2 | 0.33 | No |
| 27 | 3983 | MICU | 18 | 8 | 5 | 0.63 | Yes |
| 28 | 7724 | MICU | 10 | 7 | 3 | 0.43 | Yes |
| 29 | 3677 | Both | 12 | 8 | 2 | 0.25 | No |
| 30 | 8186 | Both | 22 | 8 | 2 | 0.25 | Yes |
| 31 | 13666 | MICU | 14 | 6 | 4 | 0.67 | Yes |
| 32 | 5874 | Both | 12 | 6 | 2 | 0.33 | No |
| 33 | 9342 | Both | 10 | 6 | 3 | 0.50 | No |
| 34 | 6045 | SICU | 8 | 5 | 2 | 0.40 | Yes |
| 35 | 7029 | MICU | 12 | 7 | 4 | 0.57 | Yes |
| 36 | 2906 | SICU | 8 | 5 | 2 | 0.40 | Yes |
| 37 | 8639 | MICU | 10 | 6 | 2 | 0.33 | Yes |
| 38 | 7437 | Both | 12 | 6 | 3 | 0.50 | No |

Description of the ICUs included in the study

N: number of patients included in the PRE and POST periods. MICU: medical ICU; SICU: surgical ICU; Both: polyvalent ICU. The number of beds is the mean averaged over the period of inclusion.

Members of the CUB-REA Group

The following individuals are members of the CUB-REA Group: **P. Trouiller**  (Hôpital Antoine Béclère, Clamart), **J-P. Bedos** (Hôpital André Mignot, Versailles), **A. Vieillard-Baron** (Hôpital Ambroise Paré, Boulogne), **Y. Cohen** (Hôpital Avicenne, Bobigny), **J. Labrousse** (Hôpital Boucicaut, Paris), **M. Safar** (Hôpital Broussais, Paris), **C. Richard** (Hôpital du Kremlin-Bicêtre, Kremlin- Bicêtre), **M. Wolff** (Hôpital Bichat, Paris), **G. Choukroun** (Centre Hospitalier Sud-Francilien), **J.-P. Mira** (Hôpital Cochin, Paris), **B. Verdière** (Hôpital Delafontaine, Saint-Denis), **P. Kalfon** (Hôpital des Diaconesses, Paris), **J.-Y. Fagon** (Hôpital Européen Georges Pompidou, Paris), **P. Ho** (Hôpital de Gonesse, Gonesse), **C. Brun-Buisson** (Hôpital Henri Mondor, Créteil), **A. Rabbat** (Hôpital Hôtel-Dieu, Paris), **F. Blot** (Institut Gustave Roussy, Villejuif), **C. Baillard** (Hôpital Jean Verdier, Bondy), **D. Dreyfus** (Hôpital Louis Mourier, Colombes), **F. Baud** (Hôpital Lariboisière, Paris), **C. Bornstain** (Hôpital Intercommunal Le Raincy-Montfermeil), **L. Pallot** (Hôpital André Grégoire, Montreuil), **H. Bismuth** (Hôpital Paul Brousse, Villejuif), **T. Similowski** (Hôpital Pitié-Salpétrière, Paris), **J. Chastre** (Hôpital Pitié-Salpétrière, Paris), **F. Bolgert** (Hôpital Pitié -Salpétrière, Paris), **J.-L. Ricome** (Centre Hospitalier Intercommunal Poissy-Saint-Germain-en-Laye), **H. Outin** (Centre Hospitalier Intercommunal Poissy-Saint-Germain-en-Laye), **F. Santoli** (Hôpital Robert Ballanger, Aulnay sous Bois), **D. Annane** (Hôpital Raymond Poincaré, Garches), **B. Guidet** (Hôpital Saint-Antoine, Paris), **B. Misset** (Hôpital Saint-Joseph, Paris), **B. Schlemmer** (Hôpital Saint-Louis, Paris), **L. Jacob** (Hôpital Saint-Louis, Paris), **M. Fartoukh** (Hôpital Tenon, Paris), F. Bonnet (Hôpital Tenon, Paris), and **H. Mentec** (Hôpital Victor Dupouy, Argenteuil).
